# Supplementary material for: Pan-Antarctic analysis aggregating spatial estimates of Adélie penguin abundance reveals robust dynamics despite stochastic noise
Source: Nat Commun. 2017 Oct 10;8:832. doi: 10.1038/s41467-017-00890-0 (PMC5635117; doi:10.1038/s41467-017-00890-0)
Supplement: Supplementary file 2 — Description of Additional Supplementary Files [file 41467_2017_890_MOESM2_ESM.docx]

**Description of Additional Supplementary Files**

File Name: Supplementary Data 1

Description: Complete details on the Adélie population model (ver. 1.2 in www.penguinmap.com), including how observations errors were incorporated into the model, a description of the model and the hindcasting methodology, details regarding the model’s winter and summer sea ice covariates, and the model’s joint distribution, additional assumptions, derived quantities, and validation.

File Name: Supplementary Data 2

Description: Complete details on the implementation of the Adélie population model (ver. 1.2 in www.penguinmap.com) in JAGS, including JAGS code, summary table of model coefficients, trace plots, convergence diagnostics, and Bayesian posterior predictive check.

File Name: Supplementary Data 3

Description: R and SQL code used to query MAPPPD’s penguin population PostgreSQL database publicly available on Amazon RDS and convert these tables into R objects used in the Adélie population model (ver. 1.2 in www.penguinmap.com).

File Name: Supplementary Data 4

Description: Exploratory analysis of correlations between the stochastic year-effects from the Adélie population model (ver. 1.2 in www.penguinmap.com) and environmental covariates hypothesized to influence penguin population dynamics.

File Name: Supplementary Data 5

Description: Exploratory analysis of density dependence in time series from the Adélie population model (ver. 1.2 in www.penguinmap.com), including plots of actual and predicted growth rates as a function of modeled abundance for each site that had at least five annual transitions where both years contained counts.

File Name: Supplementary Data 6

Description: Simulations of process noise and trend recovery, including a description of the simulation procedure, the R function used to compute the proportion of times the sign of the true population growth rate, , was unambiguously recovered from time series of varying lengths (years) summed across varying numbers of sites, given a chosen population growth rate and process error, and heat maps showing outcomes from the simulations for different growth rates and process errors consistent with the Adélie population model.

File Name: Supplementary Data 7

Description: Continental-wide model outcomes from the Adélie population model (ver. 1.2 in www.penguinmap.com), including a histogram showing the number of seasons of actual data for all sites in the model, a time series of modeled nest abundances aggregated to the continental scale, actual and predicted average population growth rate multipliers for each breeding site, and site and year specific breeding productivities for all site and year combinations where chick counts were included in the model.

File Name: Supplementary Data 8

Description: Site-specific model outcomes from the Adélie population model (ver. 1.2 in www.penguinmap.com) for the Antarctic Peninsula region (CCAMLR sub-areas 48.1, 48.2), including a histogram showing the number of seasons of actual data for all sites in the region, a data matrix showing for which years MAPPPD contained nest and/or chick counts for each site in the region, a time series of modeled nest abundances aggregated to the regional scale, time series of average peak winter and summer sea ice conditions at the regional scale, and site-level time series showing modeled nest abundance and observed and predicted nest and chick counts.

File Name: Supplementary Data 9

Description: Site-specific model outcomes from the Adélie population model (ver. 1.2 in www.penguinmap.com) for Western Antarctica (CCAMLR sub-areas 88.1, 88.2, 88.3), including a histogram showing the number of seasons of actual data for all sites in the region, a data matrix showing for which years MAPPPD contained nest and/or chick counts for each site in the region, a time series of modeled nest abundances aggregated to the regional scale, time series of average peak winter and summer sea ice conditions at the regional scale, and site-level time series showing modeled nest abundance and observed and predicted nest and chick counts.

File Name: Supplementary Data 10

Description: Site-specific model outcomes from the Adélie population model (ver. 1.2 in www.penguinmap.com) for Eastern Antarctica (CCAMLR sub-areas 58.4.1, 58.4.2), including a histogram showing the number of seasons of actual data for all sites in the region, a data matrix showing for which years MAPPPD contained nest and/or chick counts for each site in the region, a time series of modeled nest abundances aggregated to the regional scale, time series of average peak winter and summer sea ice conditions at the regional scale, and site-level time series showing modeled nest abundance and observed and predicted nest and chick counts.
