# Supplementary material for: Pan-Antarctic analysis aggregating spatial estimates of Adélie penguin abundance reveals robust dynamics despite stochastic noise
Source: Nat Commun. 2017 Oct 10;8:832. doi: 10.1038/s41467-017-00890-0 (PMC5635117; doi:10.1038/s41467-017-00890-0)
Supplement: Supplementary file 3 — Supplementary Data 1 [file 41467_2017_890_MOESM3_ESM.html]

#### Supplementary Data 1

Complete details on the Adélie population model (ver. 1.2 in www.penguinmap.com).

---

#### Table of Contents

I. Introduction

II. Observation error

III. Observation process

IV. Environmental covariates

V. Abundance process

VI. Hindcasting explained

VII. Occupancy process

VIII. Joint distribution

IX. Additional assumptions

X. Model validation

XI. Derived quantities

XII. References

---

#### I. Introduction

We used a Bayesian paired state-space abundance model and occupancy model to estimate annual Adélie abundances and probabilities of occupancy for all 267 known Adélie breeding sites from 1982 – 2016.

#### II. Observation error

Counts were provided by the observer(s) as the nest or chick count \(y\) along with an associated accuracy score (which we convert to measurement error) such that \(y\) represented a draw from a distribution:

\[\big[\,y \mid z, \sigma^{2}\_{\textrm{observation}} \,\big]\textrm{,}\]

centered on the “true” count \(z\) whose dispersion was controlled by \(\sigma^{2}\_{\textrm{observation}}\), which represented the uncertainty in the count due to measurement imprecision. The accuracy scores were selected from a 5 point scale that penguin census counters traditionally use to represent count precision1. Based on this scale, if 100 nests was the true count then the 95% confidence intervals for each accuracy category’s distribution was defined as:

Table S1-1: Reported accuracy categories and their confidence intervals.

| Reported accuracy | Reported 95% confidence interval |
| --- | --- |
| 1 | (95, 105) |
| 2 | (90, 110) |
| 3 | (75, 125) |
| 4 | (50, 150) |
| 5 | (40, 245) |

We used the lognormal distribution to model the observation process (see section III below), as all counts must be positive. However, the confidence intervals (with the exception of category 5) were symmetric around the true count and did not correspond to the skewed credible intervals generated by the lognormal distribution. To compute the appropriate scale parameters for each accuracy category, we defined a function `min.RSS` that outputed the squared deviations between the upper and lower confidence interval (Table 1) and the 0.975 and 0.025 quantiles from the cumulative density function for a lognormal distribution whose median was 100. We then used the `optim` function to select the scale parameter \(\sigma\_{\textrm{observation}}\) that minimized the sums of squares for each accuracy category. For example:

```
min.RSS <- function(par = 0.02, start = 95, end = 105) {
  (qlnorm(0.9775, log(100), par) - end)^2 + (qlnorm(0.0225, log(100), par) - start)^2
}
optim(par = 0.02, min.RSS, method = "Brent", lower = 1e-04, upper = 10)
```

Using the scale parameters, we computed 95% confidence intervals from lognormal distributions whose median were 100, i.e. the location parameters \(\mu\) = log(100). To do this, we converted each scale parameter into its geometric standard deviations \((s^{\*}=e^{\sigma\_{\textrm{observation}}})\) and calculated the 95% confidence interval as \((z(s^{\*})^{-2}, z(s^{\*})^{2})\). The scale parameters used in the Adélie observation model, their geometric standard deviations, and the adjusted 95% confidence intervals for each accuracy category were:

Table S1-2: Reported accuracy categories mapped to lognormal scale parameters used in the Adélie model.


| Reported accuracy | Reported 95% confidence interval | Scale parameter \((\sigma\_{\textrm{observation}})\) | Geometric SD | Adjusted 95% confidence interval |
| --- | --- | --- | --- | --- |
| 1 | (95, 105) | 0.025 | 1.03 | (95, 105) |
| 2 | (90, 110) | 0.050 | 1.05 | (91, 110) |
| 3 | (75, 125) | 0.120 | 1.12 | (79, 127) |
| 4 | (50, 150) | 0.230 | 1.25 | (64, 156) |
| 5 | (40, 245) | 0.510 | 1.56 | (41, 245) |

#### III. Observation process

We modeled nest or chick counts \(\,y\_{s,y,v}\) in the \(v\_{th}\) visit in the \(y\_{th}\) year at the \(s\_{th}\) site:

\[\textrm{lognormal} \big(y\_{s,y,v} \mid f\big(\theta \big), \sigma^{2}\_{\_{\textrm{observation}} s,y,v} \big)\textrm{,}\]

where

\[f\big(\theta \big) = \log\big(\alpha\_{s,y}\big)\textrm{chicks}\_{s,y,v} + \log\big(z\_{s,y}\big)\textrm{.}\]

We specified the median of the lognormal distribution to be \(z\_{s,y}\) (in the case of nest counts) or \(\alpha\_{s, y}\cdot z\_{s,y}\) (in the case of chick counts), where \(z\_{s,y}\) represents the “true” nest abundance, \(\alpha\_{s,y}\) represents the number of Adélie chicks produced per nest that survive to creche (breeding productivity), and `chicks` was a binary indicator variable equaling one if the count was a chick count and zero otherwise. We modeled breeding productivity hierarchically as:

\[\textrm{truncated normal} \big( \alpha\_{s,y} \mid \gamma, \sigma^{2}\_{\textrm{breeding}}, 0, 2 \big)\textrm{,}\] where \(\sigma\_{\textrm{breeding}}^{2}\) reflected the variation in breeding productivity due to environmental and/or demographic stochasticity. For each visit, the observation error was initially provided by the observer in the form of an accuracy rating which we then converted to its associated standard deviation \(\sigma\_{\_{\textrm{observation}} s,y,v}\) using the process outlined in Section II above. In the case of multiple visits to a site in a given year, we selected the maximum nest count and/or the maximum chick count. In this way, a site can have both a nest and chick count but never more than one of each.

#### IV. Environmental covariates

We chose covariates hypothesized to influence Adélie breeding success that were available for all sites and years included in this study. Krill abundance relies on winter sea ice conditions, with krill populations requiring at least one year of heavy winter sea ice every 4-5 years to replenish standing stocks2. Based on this, we defined peak winter sea ice concentration (`wsic`) in the Adélie model as the maximum monthly concentration (June through September) across the previous 5 winters in a 500 km radius around each Adélie site. This covariate was standardized and has a mean and standard deviation of 81 and 12, respectively.

Summer sea ice conditions can increase Adélie energy expenditures per foraging bout, and can lead to nest abandonment or skip breeding3. Since many Adélies return to their natal sites as first time breeders at age four4, we defined summer sea ice concentration (`ssic`) in the Adélie model as the maximum monthly concentration (November through January) in a 500 km radius around each Adélie site 4 years prior to the current season. This covariate was standardized and has a mean and standard deviation of 53 and 26, respectively.

Monthly sea-ice concentration (percent of sea surface covered by ice) was obtained from the satellite-based Nimbus 7 SMMR, and SSM/I-SSMIS passive microwave sensors from 1995-2015, processed by the NASA Team algorithm5 via the National Snow and Ice Data Center6. The correlation coefficient between `wsic` and `ssic` was 0.84. In order to predict Adélie abundance in 2016 for all 267 sites, we averaged each site’s 2011 - 2015 peak winter and summer sea ice as 2016 sea ice conditions are not yet publicly available.

Commercial krill fishing could have a negative effect on food resources available to Adélies during the breeding season. Alternatively, krill harvest could be positively associated with Adélie abundance if fishing effort was positively correlated with krill abundance. While the relationship between krill fishing and Adélie growth rate would be of interest for many working in the Antarctic community, we do not have data on either krill catch or krill densities with the spatial resolution that would be required to link meaningfully with site level growth rates.

#### V. Abundance process

We modeled “true” or latent nest counts \(z\_{s,y}\) at the \(s\_{th}\) site in the \(y\_{th}\) year as:

\[\textrm{lognormal} \big(z\_{s,y} \mid \log\big(g\big(\theta\big)\big) - \frac{\sigma^{2}\_{\textrm{process}}}{2}, \sigma^{2}\_{\textrm{process}} \big)\textrm{,}\]

where \(\sigma^{2}\_{\textrm{process}}\) represented process noise7, or the variation in true abundance driven by unmodeled biotic or abiotic processes not captured in \(g\big(\theta\big)\). We specified the mean of the lognormal distribution (Eq. 4) to be an exponential growth model where Adélie abundance at the \(s\_{th}\) site in the \(y\_{th}\) year was the product of Adélie abundance in the previous year and the exponentiated population growth rate \((r)\):

\[
\begin{align}
g\big(\theta\big) = z\_{s,y-1}e^{r\_{s,y}}\textrm{.}
\end{align}
\] We modeled the intrinsic growth rate \(r\) as a linear function of winter and summer sea ice concentration, continent-wide year effects, and individual site effects: \[
\begin{align}
r\_{s,y} = \beta\_{1} + \beta\_{2}\textrm{wsic}\_{s,y} + \beta\_{3}\textrm{ssic}\_{s,y} + \epsilon\_{y}
\end{align}
\] It is important to note that the sum of the medians of lognormally distributed random variables does not equal the median of the sums, making median regional abundances biologically unreasonable when the site abundances comprising these sums are non-symmetric8. To avoid this issue, we followed8 and moment matched \(g\big(\theta\big)\) to the mean, as opposed to the median, of the lognormal distribution:

\[\begin{align}
g\big(\theta\big) &= e^{\,\mu \,+\, \frac{\sigma^{2}\_{\textrm{process}}}{2}}\textrm{,}\\
\log \big(g\big(\theta\big)\big) &= \mu + \frac{\sigma^{2}\_{\textrm{process}}}{2}\textrm{,}\\
\mu &= \log \big(g\big(\theta\big)\big) - \frac{\sigma^{2}\_{\textrm{process}}}{2}\textrm{.}
\end{align}\]

We modeled year effects hierarchically as:

\[\textrm{normal} \big( \epsilon\_{y} \mid 0, \sigma^{2}\_{\textrm{year}} \big)\textrm{,}\]

where \(\sigma\_{\textrm{year}}^{2}\) reflected the variation in seasonal effects due to environmental conditions not associated with winter or summer sea ice concentration or, less likely, large scale demographic stochasticity. The distribution by CCAMLR subarea varied annually for sites with count data, with the majority of sites coming from 48.1 and 88.1 in almost all years (Fig. S1-1). We note that roughly 75% of all Adélie penguins breed in either 48.1 or 88.1, so these data rich sites also happen to be the ones with the majority of Adélies; as such, their considerable influence on the model was neither unexpected nor inappropriate.

Fig. S1-1: Breakdown by CCAMLR subarea of sites with count data in a given year.

#### VI. Hindcasting explained

We hindcast “true” or latent nest counts \(z\_{s,y}\) for the \(s\_{th}\) site beginning with the year prior to its initial year of data \((I\_{s})\) and iterating backwards to 1982 as:

\[\textrm{lognormal} \big(z\_{s,y-1} \mid \log\big(g^{-1}\big(\theta\big)\big) - \frac{\sigma^{2}}{2}, \sigma^{2}\_{\textrm{process}} \big)\textrm{,}\] where the mean of the lognormal distribution was:

\[g^{-1}\big(\theta\big) = \frac{z\_{s,y}}{e^{\,\beta\_{1} \,+\, \beta\_{2}\textrm{wsic}\_{s,y} \,+\, \beta\_{3}\textrm{ssic}\_{s,y} \,+\, \epsilon\_{y}}}\textrm{.}\] This method of hindcasting was possible due to the fact that the exponential growth function can be inverted:

\[\begin{align}
z\_{s,y} &= z\_{s,y-1}e^{r}\textrm{,}\\
z\_{s,y-1} &=\frac{z\_{s,y}}{e^{r}}\textrm{,}\\
\end{align}\]

making hindcast nest counts functionally no different than forecasted nest counts at the end of a site’s time series or in years of missing data within a site’s time series. For sites whose first year of data was 1982, no hindcasting was necessary.

#### VII. Occupancy process

Given that the detection rate for a known penguin breeding site approaches 1.0 (unpublished), we modeled “true” site occupancy \(w\_{s,y}\) at the \(s\_{th}\) site in the \(y\_{th}\) year directly as:

\[\textrm{Bernoulli} \big( w\_{s,y} \mid h\big(\theta\big)\big)\textrm{,}\]

where

\[h\big(\theta\big) = \cfrac{w\_{s,y-1}}{1+e^{\phi}}\textrm{,}\]

and \(\phi\) represented site persistence, or the probability that a site occupied in year \(y\) was occupied in year \(y+1\). Based on the data included in the Adélie model, we assumed no Adélie site colonization from 1982 – 2014 and allowed sites to either persist or go extinct without the possibility of future recolonization. The site occupancy data in the Adélie model \((w\_{s,y})\) was collapsed abundance data supplemented with a small number of presence only observations. We replaced missing values bracketed by observed presences:

\[\big[\ldots, 1, \textrm{NA}, 1, \ldots \big]\textrm{,}\]

with \(w=1\) as Adélie colonization was assumed to be impossible.

#### VIII. Joint distribution

The joint distribution for the Adélie model was:

\[\begin{align}
\big[ \boldsymbol{\alpha}, \boldsymbol{\beta}, \gamma, \boldsymbol{\epsilon}, \phi, \sigma\_{\textrm{process}}, \sigma\_{\textrm{year}}, \sigma\_{\textrm{breeding}}, \mathbf{z} \mid \mathbf{y}, \boldsymbol{\sigma\_{\textrm{observation}}}, \mathbf{w} \big] \varpropto & \\
& \prod\_{s=1}^{267} \prod\_{y\,\in\,Y\_{s}} \prod\_{v=1}^{V\_{s,y}} \textrm{lognormal} \big( y\_{s,y,v} \mid f\big( \theta \big), \sigma\_{\_{\textrm{observation}}s,y,v}^{2} \big) \nonumber \\
& \times \prod\_{s=1}^{267} \prod\_{y\,=\,I\_{s}\,+\,1}^{35} \textrm{lognormal} \big( z\_{s,y} \mid \log \big(g\big(\theta\big)\big) - \frac{\sigma^{2}\_{\textrm{process}}}{2}, \sigma^{2}\_{\textrm{process}} \big) \nonumber \\
& \times \prod\_{s=1}^{267} \prod\_{y\,=\, 1}^{I\_{s}-1} \textrm{lognormal} \big( z\_{s,y} \mid \log \big(g^{-1}\big(\theta\big)\big) - \frac{\sigma^{2}\_{\textrm{process}}}{2}, \sigma^{2}\_{\textrm{process}} \big) \nonumber \\
& \times \prod\_{s=1}^{267} \prod\_{y\,=\, 1}^{35} \textrm{Bernoulli} \big( w\_{s,y} \mid h\big(\theta \big) \big)\, \textrm{uniform} \big( z\_{s,\,I\_{s}} \mid 0, 10^{6} \big) \nonumber \\
& \times \textrm{normal} \big( \epsilon\_{y} \mid 0, \sigma^{2}\_{\textrm{year}} \big) \, \textrm{truncated normal} \big( \alpha\_{s, y} \mid \gamma, \sigma^{2}\_{\textrm{breeding}}, 0, 2 \big) \nonumber \\
& \times \prod\_{i=1}^{3} \textrm{normal} \big( \beta\_{i} \mid 0, 100 \big)\, \textrm{uniform}\big(\phi \mid 0.1, 1\big) \nonumber \\
&\times \textrm{uniform} \big( \sigma\_{\textrm{process}} \mid 0, 1 \big) \, \textrm{uniform} \big( \sigma\_{\textrm{year}} \mid 0, 1 \big) \nonumber \\
& \times \textrm{gamma} \big( \gamma \mid \frac{0.89^{2}}{0.05^{2}}, \frac{0.89}{0.05^{2}} \big) \, \textrm{gamma} \big( \sigma\_{\textrm{breeding}} \mid \frac{0.42^{2}}{0.05^{2}}, \frac{0.42}{0.05^{2}} \big) \textrm{,} \nonumber \\
\end{align}\]

where \(Y\_{s}\) was the set of years with nest and/or chick counts for the \(s\_{th}\) site and \(I\_{s}\) was the first year a count was made at the \(s\_{th}\) site. The lower bound of the prior distribution for \(\phi\) was restricted to 0.1 to aid convergence and because penguin colonies rarely go extinct. We used informed priors derived from9 for the mean \(\gamma\) and standard deviation \(\sigma\_{\textrm{breeding}}\) of the truncated normal distribution from which site-year breeding productivities \(\alpha\_{s,y}\) were drawn. We chose endpoints to this truncated distribution that spanned the possible range of penguin productivity. All other parameters were given vague priors.

#### IX. Additional assumptions

1. We excluded one Avian Island count which had a very large discrepancy from the other counts at that site during similar time period.
2. We converted adults counts 1:1 to nest counts prior to modeling. We recognize that for any individual survey, this may not be the correct conversion factor. Adult counts may be reported when surveys are done very early in the season (prior to nesting), when nests have not yet been established and the count of adults is too small. Alternatively, surveys may be conducted at a time when more than one adult is in attendance at some nests and the count of adults is too high. Because this conversion introduces an additional uncertainty in the conversion to nests, we increased the error category of adult counts by + 3 (with a max error of 5) relative to the reported uncertainty in the adult count itself. This approach is quite conservative, in that we likely overestimate the true uncertainty of those adult counts. Additionally, it is worth noting that the adult counts in our updated database come overwhelmingly (86%) from time series (published by10 and others) comprised only of adult counts, so any bias (separate from the uncertainty) in this conversion factor (between adults and nests) will not affect our inference regarding the dynamics at these sites, nor the relationship between population growth rate and environmental drivers. Fortunately, the trends observed at these sites are unambiguous (see site-level time series for BENT, HOKU, MAME, MIZU, NOKK, ONGU, RUMP, TOKI, and YTRE in Supplementary Data 10), and given that the time series from10 are all relatively complete, we can’t imagine a scenario where these trends would emerge purely as an artifact of variable survey timing. We note that our findings are not only consistent with, but recapitulate the interpretation of these data as presented in10. NB: Several of the authors of this paper (Lynch, Jenouvrier, Youngflesh, McDowall) are involved in another project in which UAVs were used to survey both adults and nest counts at the peak of nesting, and we find that a count of adults are only 5-20% larger than a count of occupied nests. While this is no guarantee that the adult-to-nest bias inherent to those included in our model are similarly small, it does suggest that an appropriately timed adult count is closely related to the number of nesting pairs and so the impact on our estimate of abundance (as an absolute measure) in that region will be minimal.
3. We excluded absence counts made via very high resolution satellite imagery, since the detection function for small colonies remains an area of active research.
4. There were 23 instances where a site’s initial count was solely a chick count. We converted these directly nest counts prior to fitting the Adélie model by dividing them by the mean breeding productivity (0.9 chicks/nest) extracted from9 in order to avoid convergence issues for the initial year breeding productivities \(\alpha\_{s,y}\) at these sites.

#### X. Model validation

We performed out-of-sample validation to assess the Adélie model’s predictive performance. We refit the model after randomly withholding ~10% of the Adélie counts. The validation dataset included both chick (20.6%) counts and nest counts (79.4%) collected from the Antarctic Peninsula (42.8%), western Antarctica (30.5%), and eastern Antarctic (26.7%). We estimated the posterior predictive distributions for all withheld counts. Uncertainty in these predictive distributions could arise from three sources: 1) uncertainty in the true nest abundance and/or 2) uncertainty in the breeding productivity (in the case of chick counts), and 3) observer measurement error. While these combined sources of uncertainty resulted in several very large predictive intervals, 25% of the predictive 95% credible intervals were \(<1000\) chicks/nests and 52% of these intervals \(\leq 10000\) chicks/nests. These predictive intervals were small enough relative to Adélie colony sizes to be biologically meaningful. We determined that 96% of the nest/chick counts fell within the predictive 95% credible intervals for the withheld counts, indicating that the Adélie model has good predictive performance.

#### XI. Derived quantities

We computed the following derived quantities:

1. The state of the \(s\_{th}\) site in the \(y\_{th}\) year as \(z^{\*}\_{s,y}=z\_{s,y}\cdot w\_{s,y}\).
2. The aggregated annual abundances at the CCAMLR subarea, regional, and continent scales as the sum the posterior distributions of \(z^{\*}\_{s,y}\) for sites within each spatial scale.
3. The predicted population growth rate multiplier \(\lambda\) for each site as \(e^{\,\beta\_{1} \,+\, \beta\_{2}\textrm{wsic}\_{s,y} \,+\, \beta\_{3}\textrm{ssic}\_{s,y} \,+\, \epsilon\_{y}}\).
4. The average actual and predicted population growth rate multipliers \(\lambda\) for each site as the geometric mean of \(\frac{z\_{s,y+1}}{z\_{s,y}}\) (actual \(\lambda\)) and \(e^{\,\mu \,+\, \beta\_{1}\textrm{wsic}\_{s,y} \,+\, \beta\_{2}\textrm{ssic}\_{s,y} \,+\, \epsilon\_{y}}\) (predicted \(\lambda\)) for all years the site was occupied between 1982 and 2015.
5. The finite population standard deviations for the effects of winter sea ice and summer sea ice as the absolute values of \(\beta\_{2}\), and \(\beta\_{3}\)11.
6. The finite population standard deviation for the year effects as the standard deviation of \(\boldsymbol{\epsilon}\)11.
7. The posterior predictive distribution for all site, year, and visit combinations for which we had nest or chick counts.

#### XII. References

1. Croxall, J. P. & Kirkwood, E. D. *The distribution of penguins on the Antarctic Peninsula and Islands of the Scotia Sea*. (British Antarctic Survey, Cambridge, United Kingdom, 1979).
2. Ainley, D. G. *et al.* Diet and foraging effort of Adélie penguins in relation to pack-ice conditions in the southern Ross Sea. *Polar Biology* **20**, 311-319 (1998).
3. Ainley, D. & Leresche, R. E. The effects of weather and ice conditions on breeding at the rookery as breeders in Adélie Penguins *Condor* **75**, 235-239 (1973).
4. Ainley, D. G. The Adélie Penguin: Bellwether of Climate Change. (Columbia University Press, New York, NY, 2002).
5. Cavalieri, D. J., Germain, K. S. & Swift, C. T. Reduction of weather effects in the calculation of sea-ice concentration with the DMSP SSM/I. *Journal of Glaciology* **41**, 455–464 (1995).
6. Cavalieri, D. J., Parkinson, C., Gloersen, P. & Zwally, H. Sea ice concentrations from Nimbus-7 SMMR and DMSP SSM/I-SSMIS Passive Microwave Data, Version 1 1978-2014. NASA National Snow and Ice Data Center Distributed Active Archive Center. http://dx.doi.org/10.5067/8GQ8LZQVL0VL (2016).
7. Ahrestani, F. S., Hebblewhite, M. & Post, E. The importance of observation versus process error in analyses of global ungulate populations. *Scientific Reports* **3**, 3125 (2013).
8. Methot, Jr., R. D. & Taylot, I. G. Adjusting for bias due to variability of estimated recruitments in fishery assessment models. *Canadian Journal of Fisheries and Aquatic Sciences* **68**, 1744-1760 (2011).
9. Youngflesh, C. *et al.* Circumpolar analysis of the Adélie penguin reveals the importance of environmental variability in phenological mismatch. *Ecology* **98**, 940-951 (2017).
10. Kato, A. & Ropert-Coudert, Y. Rapid increase in Adélie penguin populations in Lützow-Holm Bay area since the mid 1990s. *Polar Bioscience* **20**, 55–62 (2006).
11. Gelman, A. & Hill, J. *Data Analysis Using Regression and Multilevel/Hierarchical Models*. (Cambridge University Press, New York, New York, 2007).
