# Supplementary material for: Pan-Antarctic analysis aggregating spatial estimates of Adélie penguin abundance reveals robust dynamics despite stochastic noise
Source: Nat Commun. 2017 Oct 10;8:832. doi: 10.1038/s41467-017-00890-0 (PMC5635117; doi:10.1038/s41467-017-00890-0)
Supplement: Supplementary file 4 — Supplementary Data 2 [file 41467_2017_890_MOESM4_ESM.html]

#### Supplementary Data 2

Complete details on the implementation of the Adélie population model (ver. 1.2 in www.penguinmap.com) in JAGS, including JAGS code, summary table of model coefficients, trace plots, convergence diagnostics, and Bayesian posterior predictive check.

---

#### Table of Contents

I. Introduction

II. Adélie model in JAGS

III. JAGS run

IV. Initial values, summary statistics, and trace plots

V. Gelman-Rubin diagnostic

VI. Posterior predictive check

VII. References

---

#### I. Introduction

We estimated the posterior distributions for all parameters and latent states in the Adélie model using Monte Carlo methods (MCMC) implemented in JAGS 4.1 with the rjags package in R 3.3.1 on Amazon’s Elastic Compute Cloud (Amazon EC2)1,2. We computed three chains for each random variable, each with different initial values. After a burnin period of 300,000 iterations, we accumulated 15,000 samples from each chain, keeping every 10th sample. We assessed convergence through visual inspection of trace plots and using the Gelman-Rubin diagnostic3. We verified that the model adequately captured the dispersion in nest counts (measured as the sum of the squared residuals) using posterior predictive checks3. We confirmed that the Bayesian p-value, defined as the probability that the simulated data were more extreme than the observed data, was indicative of a good model fit4. We purposefully retained both sea ice covariates regardless of their effect sizes. We justify this approach by noting that the covariates were selected intentionally as part of our original survey design to test long-standing biological hypotheses regarding how sea ice concentration impacts Adélie population dynamics. We report the medians and 95% highest posterior density or equal-tailed credible intervals of all modeled coefficients and derived quantities, unless otherwise noted.

#### II. Adélie model in JAGS

```
{
sink("MAPPPDModel.jags")
cat("

model {
  
################################################################################################################
# priors

###### process and season variance priors
for (i in 1:2) {
  tau[i] <- pow(sigma[i], -2)
  sigma[i] ~ dunif(0, 1)
}

###### sea ice priors
for (i in 1:3) {
  beta[i] ~ dnorm(0, 1e-04)
}

###### chick conversion priors
# mu of truncated normal, .89, .05
# sigma of truncated normal, .42, .05

a[1] <- .89^2/.05^2
b[1] <- .89/.05^2
a[2] <- .42^2/.05^2
b[2] <- .42/.05^2

gamma ~ dgamma(a[1], b[1])
sigma[3] ~ dgamma(a[2], b[2])
tau[3] <- pow(sigma[3], -2)

##### occupancy prior
phi ~ dunif(0.1, 1)

################################################################################################################
# year effects for nest abudance and breeding success effects

###### season effects
for (t in 1:n_seasons) {

  epsilon[t] ~ dnorm(0, tau[1])
}

###### breeding success

for (i in 1:n_sites) {
  for (t in 1:n_seasons) {

    alpha[i, t] ~ dnorm(gamma, tau[3])T(0, 2)

  }
}

################################################################################################################
# intial year abundance and growth rate

for (i in 1:n_sites) {

  zpeak[i, initial[i]] ~ dunif(1, 1e+06)
  zr[i, initial[i]] <- beta[1] + beta[2] * wsic[i, initial[i]] + beta[3] * ssic[i, initial[i]] + epsilon[initial[i]]

}

################################################################################################################
# abundance process model: intial year + 1: year 35 (2016)

for (i in 1:n_sites) {
  for (t in (initial[i] + 1):n_seasons) {

    zpeak[i, t] ~ dlnorm(mu_zpeak[i, t], tau[2])
    mu_zpeak[i, t] <- log(zpeak[i, t - 1] * exp(zr[i, t])) - 1/(2 * tau[2])
    zr[i, t] <- beta[1]  + beta[2] * wsic[i, t] + beta[3] * ssic[i, t] + epsilon[t]

  }
}

################################################################################################################
# abundance process model: year 1 (1982): intial year - 1

for (i in 1:n_sites) {
  for (t in 1:(initial[i] - 1)) {

    zpeak[i, initial[i] - t] ~ dlnorm(med_zpeak[i, initial[i] - t], tau[2])
    med_zpeak[i, initial[i] - t] <- log(zpeak[i, initial[i] - t + 1]/exp(zr[i, initial[i] - t + 1])) - 1/(2 * tau[2])
    zr[i, t] <- beta[1] + beta[2] * wsic[i, t] + beta[3] * ssic[i, t] + epsilon[t]

  }
}

################################################################################################################
# occupancy model

for (i in 1:n_sites) {
  for (t in 2:n_seasons) {

    w[i, t] ~ dbern(mu_w[i, t])
    mu_w[i, t] <- w[i, t - 1] * phi

  }
}

################################################################################################################
# observation model

for (i in 1:n) {

  y[site[i], season[i], visit[i]] ~ dlnorm(med_y[site[i], season[i], visit[i]], precision[site[i], season[i], visit[i]])
  med_y[site[i], season[i], visit[i]] <- chick[site[i], season[i], visit[i]] * log(alpha[site[i], season[i]]) + log(zpeak[site[i], season[i]])

}

################################################################################################################
# prediction for data validation

#for (i in 1:n_p) {

#  y_p[i] ~ dlnorm(med_y[site_p[i], season_p[i], visit_p[i]], precision[site_p[i], season_p[i], visit_p[i]])
#  med_y[site_p[i], season_p[i], visit_p[i]] <- chick[site_p[i], season_p[i], visit_p[i]] * log(alpha[site[i], season[i]]) + log(zpeak[site_p[i], season_p[i]])

#}

################################################################################################################
# posterior predictive check

for (i in 1:n) {

  ynew[i] ~ dlnorm(med_y[site[i], season[i], visit[i]], precision[site[i], season[i], visit[i]])
  ysqActual[i] <- pow((y[site[i], season[i], visit[i]] - exp(med_y[site[i], season[i], visit[i]])), 2)
  ysqNew[i] <- pow((ynew[i] - exp(med_y[site[i], season[i], visit[i]])), 2)

}

zActual <- sum(ysqActual[])
zNew <- sum(ysqNew[])

################################################################################################################
# derived quantities: zstate and geometric means

for (i in 1:n_sites) {
  for (t in 1:n_seasons) {
    zstate[i, t] <- w[i, t] * zpeak[i, t]
  }
}

for (i in 1:n_sites) {
  for (t in 2:(n_seasons - 1)) {

    zlambdaActual[i, t - 1] <- ifelse(w[i, t] == 1, zpeak[i, t]/zpeak[i, t - 1], 1)
    zlambdaPredicted[i, t - 1] <- ifelse(w[i, t] == 1, exp(zr[i, t]), 1)

  }
}

for (i in 1:n_sites) {

  zgmeanActual[i] <- pow(prod(zlambdaActual[i, ]), (1/sum(w[i, 2:(n_seasons - 1)])))
  zgmeanPredicted[i] <- pow(prod(zlambdaPredicted[i, ]), (1/sum(w[i, 2:(n_seasons - 1)])))

}

################################################################################################################
# derived quantities: finite population sd 

sd_season <- sd(epsilon[])
sd_wsic <- abs(beta[2])
sd_ssic <- abs(beta[3])

}
",fill = TRUE)
sink()
}
```

#### III. JAGS run

```
n.adapt <- 5000
n.update <- 300000
n.iter <- 15000
parms <- c(
  "beta", 
  "gamma",
  "sigma",
  "phi", 
  "epsilon", 
  "alpha",
  "zpeak",
  "w",
  "zstate", 
  "zr",
  "sd_season", 
  "sd_wsic", 
  "sd_ssic", 
  "zgmeanActual", 
  "zgmeanPredicted", 
  "zActual", 
  "zNew",
  "ynew")
```

```
cl <- makeCluster(3)

pid <- NA
for(i in 1:3){
 pidNum <- capture.output(cl[[i]])
 start <- regexpr("pid", pidNum)[[1]]
 end <- nchar(pidNum)
 pid[i] <- substr(pidNum, (start + 4), end)
}

clusterExport(cl, c("DataQuery", "finalInits", "n.adapt", "n.update", "n.iter", "parms", "n_sites", "n_seasons", "pid"))

out <- clusterEvalQ(cl, {
  
  library(rjags)
  processNum <- which(pid==Sys.getpid())
  inits <- finalInits[[processNum]]
  jm = jags.model("MAPPPDModel.jags", data = DataQuery, inits = inits, n.chains = 1, n.adapt = n.adapt)
  update(jm, n.iter = n.update)
  samples = coda.samples(jm, n.iter = n.iter, variable.names = parms, thin = 10)
  return(c(samples, inits))
  
})
```

#### IV. Initial values, summary statistics, and trace plots

```
## $beta
## [1] -0.01139230  0.02688149 -0.03855127
## 
## $sigma
## [1] 0.07545106 0.21389745 0.34923965
## 
## $gamma
## [1] 0.9389901
## 
## $phi
## [1] 0.9987208
## 
## $epsilon
##  [1] -0.2228381247 -0.0678027874 -0.0705033038 -0.0153229059 -0.0401948855
##  [6] -0.0553949374 -0.1623325433 -0.2325483779 -0.2823573056 -0.0827803790
## [11] -0.0207256051 -0.1872926204 -0.1101207131 -0.0889553729 -0.2024563290
## [16]  0.0002603336 -0.1591375972 -0.0122723683 -0.2017600193 -0.2152904735
## [21]  0.0383762610 -0.2514865568  0.0107916806 -0.0205873376 -0.2813951119
## [26] -0.1174708823 -0.1245060927  0.0339885126 -0.0829503298 -0.2181627433
## [31] -0.0577264964 -0.1324640436 -0.1206489255 -0.1322392646 -0.2195920273
## 
## $beta
## [1]  0.02595167  0.04742486 -0.01781077
## 
## $sigma
## [1] 0.1075833 0.2262457 0.3966922
## 
## $gamma
## [1] 1.00485
## 
## $phi
## [1] 0.9994676
## 
## $epsilon
##  [1]  0.001659485  0.045162480  0.027995805  0.079425863  0.051239974
##  [6]  0.039809102 -0.064076871 -0.134519631 -0.173439775  0.009989775
## [11]  0.071417854 -0.085455395 -0.001055642  0.007934432 -0.096844588
## [16]  0.102909402 -0.053140977  0.092764524 -0.092795731 -0.105467028
## [21]  0.135338069 -0.144328114  0.116064666  0.088202621 -0.167260411
## [26]  0.003176474 -0.005348048  0.142969028  0.019396862 -0.087112609
## [31]  0.066844675 -0.016460035  0.045237748  0.054010584  0.003160546
## 
## $beta
## [1] 0.070891991 0.067702206 0.003772507
## 
## $sigma
## [1] 0.1506653 0.2391844 0.4606542
## 
## $gamma
## [1] 1.071801
## 
## $phi
## [1] 0.9998366
## 
## $epsilon
##  [1]  0.2243232336  0.1640595370  0.1267334570  0.1748103101  0.1454564132
##  [6]  0.1354917927  0.0285652773 -0.0396655100 -0.0725255562  0.1083159496
## [11]  0.1662763148  0.0088467335  0.1018468890  0.1096065882  0.0006222878
## [16]  0.2063698134  0.0445720579  0.2022904320  0.0082157087  0.0014373566
## [21]  0.2416240782 -0.0361144554  0.2230605040  0.2038146568 -0.0596649883
## [26]  0.1205567374  0.1075388823  0.2616846453  0.1240940109  0.0299232028
## [31]  0.1975272572  0.0964446971  0.2233066763  0.2436821118  0.2344197261
```

```
## 
## Iterations = 305010:320000
## Thinning interval = 10 
## Number of chains = 3 
## Sample size per chain = 1500 
## 
## 1. Empirical mean and standard deviation for each variable,
##    plus standard error of the mean:
## 
##                  Mean        SD  Naive SE Time-series SE
## beta[1]      0.025102 0.0196877 2.935e-04      7.973e-04
## beta[2]      0.046732 0.0105468 1.572e-04      3.237e-04
## beta[3]     -0.016466 0.0108684 1.620e-04      3.811e-04
## sigma[1]     0.109145 0.0190458 2.839e-04      4.584e-04
## sigma[2]     0.226114 0.0065072 9.700e-05      1.902e-04
## sigma[3]     0.398186 0.0286710 4.274e-04      1.048e-03
## gamma        1.017158 0.0353876 5.275e-04      2.019e-03
## phi          0.999415 0.0002872 4.281e-06      4.280e-06
## epsilon[1]  -0.001956 0.1103096 1.644e-03      1.618e-03
## epsilon[2]   0.031602 0.0600208 8.947e-04      1.338e-03
## epsilon[3]   0.022959 0.0493535 7.357e-04      1.130e-03
## epsilon[4]   0.080818 0.0483736 7.211e-04      1.091e-03
## epsilon[5]   0.047737 0.0474767 7.077e-04      1.217e-03
## epsilon[6]   0.037912 0.0489987 7.304e-04      1.208e-03
## epsilon[7]  -0.065746 0.0491838 7.332e-04      1.274e-03
## epsilon[8]  -0.139242 0.0493295 7.354e-04      1.215e-03
## epsilon[9]  -0.176410 0.0530498 7.908e-04      1.486e-03
## epsilon[10]  0.011949 0.0492525 7.342e-04      1.337e-03
## epsilon[11]  0.070360 0.0475250 7.085e-04      1.381e-03
## epsilon[12] -0.084260 0.0486349 7.250e-04      1.436e-03
## epsilon[13] -0.001392 0.0509170 7.590e-04      1.649e-03
## epsilon[14]  0.008443 0.0501834 7.481e-04      1.494e-03
## epsilon[15] -0.096002 0.0523985 7.811e-04      1.528e-03
## epsilon[16]  0.099959 0.0524731 7.822e-04      1.695e-03
## epsilon[17] -0.055263 0.0533525 7.953e-04      1.845e-03
## epsilon[18]  0.092155 0.0540717 8.061e-04      1.713e-03
## epsilon[19] -0.090376 0.0549973 8.199e-04      1.803e-03
## epsilon[20] -0.108815 0.0547242 8.158e-04      1.696e-03
## epsilon[21]  0.140765 0.0507473 7.565e-04      1.359e-03
## epsilon[22] -0.139296 0.0555774 8.285e-04      1.812e-03
## epsilon[23]  0.119493 0.0554026 8.259e-04      1.790e-03
## epsilon[24]  0.088569 0.0577387 8.607e-04      1.726e-03
## epsilon[25] -0.168738 0.0555354 8.279e-04      1.469e-03
## epsilon[26]  0.009483 0.0623240 9.291e-04      1.923e-03
## epsilon[27] -0.003405 0.0597658 8.909e-04      2.145e-03
## epsilon[28]  0.147806 0.0586677 8.746e-04      1.861e-03
## epsilon[29]  0.022286 0.0516107 7.694e-04      1.349e-03
## epsilon[30] -0.082991 0.0625346 9.322e-04      1.598e-03
## epsilon[31]  0.071840 0.0631490 9.414e-04      1.601e-03
## epsilon[32] -0.018581 0.0580256 8.650e-04      1.356e-03
## epsilon[33]  0.050941 0.0842002 1.255e-03      1.975e-03
## epsilon[34]  0.058927 0.0927316 1.382e-03      3.172e-03
## epsilon[35] -0.001463 0.1125729 1.678e-03      1.655e-03
## 
## 2. Quantiles for each variable:
## 
##                   2.5%       25%        50%       75%      97.5%
## beta[1]     -0.0147929  0.012246  0.0252878  0.038313  0.0630782
## beta[2]      0.0262716  0.039428  0.0465041  0.053832  0.0675851
## beta[3]     -0.0378159 -0.023606 -0.0163113 -0.009288  0.0044893
## sigma[1]     0.0768228  0.095505  0.1073426  0.120953  0.1510701
## sigma[2]     0.2137411  0.221693  0.2260892  0.230311  0.2393016
## sigma[3]     0.3487998  0.378000  0.3959445  0.416473  0.4598412
## gamma        0.9472970  0.993655  1.0178858  1.040902  1.0855045
## phi          0.9987353  0.999252  0.9994580  0.999627  0.9998321
## epsilon[1]  -0.2171401 -0.073728 -0.0028399  0.066747  0.2146343
## epsilon[2]  -0.0837563 -0.008336  0.0320892  0.071371  0.1524261
## epsilon[3]  -0.0729876 -0.009719  0.0232814  0.055971  0.1202789
## epsilon[4]  -0.0150402  0.048018  0.0805699  0.113628  0.1758401
## epsilon[5]  -0.0457949  0.016275  0.0483785  0.079135  0.1412427
## epsilon[6]  -0.0584302  0.004963  0.0374427  0.070981  0.1341890
## epsilon[7]  -0.1617064 -0.098827 -0.0648917 -0.032611  0.0309420
## epsilon[8]  -0.2369588 -0.172068 -0.1400697 -0.106530 -0.0418491
## epsilon[9]  -0.2834665 -0.211689 -0.1758566 -0.140857 -0.0725786
## epsilon[10] -0.0833105 -0.022072  0.0115107  0.045252  0.1084723
## epsilon[11] -0.0219767  0.038734  0.0706077  0.102703  0.1641686
## epsilon[12] -0.1801011 -0.117592 -0.0843539 -0.050769  0.0101400
## epsilon[13] -0.1002080 -0.036194 -0.0018837  0.032728  0.0986370
## epsilon[14] -0.0933959 -0.024494  0.0091301  0.042301  0.1079871
## epsilon[15] -0.2011538 -0.130888 -0.0942979 -0.059959  0.0058005
## epsilon[16]  0.0008615  0.064430  0.0984652  0.134972  0.2033510
## epsilon[17] -0.1615985 -0.090587 -0.0560526 -0.019696  0.0484044
## epsilon[18] -0.0120081  0.056862  0.0902407  0.129283  0.1997167
## epsilon[19] -0.1980287 -0.127780 -0.0896961 -0.052796  0.0138189
## epsilon[20] -0.2168168 -0.144833 -0.1093642 -0.071489 -0.0008639
## epsilon[21]  0.0427130  0.106479  0.1397537  0.173635  0.2422492
## epsilon[22] -0.2477546 -0.177008 -0.1394304 -0.100711 -0.0304578
## epsilon[23]  0.0129773  0.081781  0.1191129  0.156067  0.2299389
## epsilon[24] -0.0255454  0.050972  0.0887647  0.127520  0.2017116
## epsilon[25] -0.2810585 -0.206098 -0.1669444 -0.130579 -0.0648342
## epsilon[26] -0.1143012 -0.033067  0.0097207  0.051627  0.1332316
## epsilon[27] -0.1211862 -0.043068 -0.0027759  0.035727  0.1157423
## epsilon[28]  0.0354799  0.108412  0.1464499  0.186933  0.2650138
## epsilon[29] -0.0794643 -0.012631  0.0221518  0.057396  0.1227361
## epsilon[30] -0.2063617 -0.124446 -0.0822930 -0.040291  0.0376857
## epsilon[31] -0.0487878  0.029969  0.0707388  0.113712  0.1971556
## epsilon[32] -0.1316206 -0.056693 -0.0183039  0.019451  0.0965324
## epsilon[33] -0.1159707 -0.004973  0.0496812  0.106530  0.2167526
## epsilon[34] -0.1191587 -0.002319  0.0576066  0.119943  0.2416638
## epsilon[35] -0.2251661 -0.076501 -0.0006493  0.072258  0.2174293
```

Fig. S2-1: Trace plots of Adélie model parameters.

#### V. Gelman-Rubin diagnostic

```
## Potential scale reduction factors:
## 
##             Point est. Upper C.I.
## beta[1]           1.01       1.01
## beta[2]           1.00       1.02
## beta[3]           1.00       1.01
## sigma[1]          1.00       1.01
## sigma[2]          1.00       1.00
## sigma[3]          1.00       1.02
## gamma             1.02       1.07
## phi               1.00       1.00
## epsilon[1]        1.00       1.00
## epsilon[2]        1.00       1.00
## epsilon[3]        1.00       1.00
## epsilon[4]        1.00       1.01
## epsilon[5]        1.00       1.00
## epsilon[6]        1.00       1.00
## epsilon[7]        1.00       1.00
## epsilon[8]        1.00       1.00
## epsilon[9]        1.00       1.02
## epsilon[10]       1.00       1.00
## epsilon[11]       1.00       1.02
## epsilon[12]       1.00       1.00
## epsilon[13]       1.00       1.00
## epsilon[14]       1.00       1.01
## epsilon[15]       1.00       1.00
## epsilon[16]       1.01       1.03
## epsilon[17]       1.01       1.02
## epsilon[18]       1.00       1.00
## epsilon[19]       1.00       1.00
## epsilon[20]       1.00       1.01
## epsilon[21]       1.01       1.03
## epsilon[22]       1.00       1.00
## epsilon[23]       1.00       1.01
## epsilon[24]       1.00       1.02
## epsilon[25]       1.00       1.00
## epsilon[26]       1.00       1.00
## epsilon[27]       1.00       1.01
## epsilon[28]       1.00       1.01
## epsilon[29]       1.00       1.00
## epsilon[30]       1.00       1.01
## epsilon[31]       1.00       1.02
## epsilon[32]       1.00       1.00
## epsilon[33]       1.00       1.01
## epsilon[34]       1.00       1.02
## epsilon[35]       1.00       1.00
```

Of the 9345 latent \(z\_{s,y}\) and \(\alpha\_{s,y}\) states, the Gelman Rubin diagnostic upper C.I. exceeds 1.1 for 2.05% of the \(z\_{s,y}\) states and 0.22% of the \(\alpha\_{s,y}\) states.

#### VI. Posterior predictive check

Fig. S2-2: Scatterplot of summed squared residuals between the observed counts (\(\,y\,\)) and the predicted median count and between simulated counts and the predicted median count at each MCMC iteration. The Bayesian posterior predictive check p-value was 0.69.

#### VII. References

1. Plummer, M. rjags: Bayesian graphical models using MCMC (2013).
2. R Development Core Team. R: A Language and Environment for Statistical Computing. R Foundation for Statistical Computing, Vienna, Austria (2016).
3. Gelman, A. & Rubin, D. B. 1992. Inference from iterative simulation using multiple sequences. *Statistical Science* **7**, 457–472 (1992).
4. Gelman, A. *et al.* *Bayesian Data Analysis*. (CRC Press, Boca Raton, Florida, 2013).
