# Supplementary material for: Pan-Antarctic analysis aggregating spatial estimates of Adélie penguin abundance reveals robust dynamics despite stochastic noise
Source: Nat Commun. 2017 Oct 10;8:832. doi: 10.1038/s41467-017-00890-0 (PMC5635117; doi:10.1038/s41467-017-00890-0)
Supplement: Supplementary file 5 — Supplementary Data 3 [file 41467_2017_890_MOESM5_ESM.html]

#### Supplementary Data 3

R and SQL code used to query the penguin population database (www.penguinmap.com) and convert these tables into R objects used in the Adélie population model (ver. 1.2 in www.penguinmap.com).

---

```
# Query MAPPPD for Adelie model data 
# Arrange Adelie data for input into jags

library("RPostgreSQL")
library("dplyr")

#############################################################################################################
# create a connection to the MAPPPD database http://www.r-bloggers.com/getting-started-with-postgresql-in-r/

rm(list = ls())

drv <- dbDriver("PostgreSQL")

mapppd.dbname <- "mapppd"
mapppd.dbhost <- "mapppd.cbmvud2go9ni.us-east-1.rds.amazonaws.com"
mapppd.dbuser <- "mapppd_public"
mapppd.dbpwrd <- "p3nguins2017"

con <- dbConnect(drv, dbname = mapppd.dbname, host = mapppd.dbhost, port = 5432, user = mapppd.dbuser, password = mapppd.dbpwrd)

#############################################################################################################
# Query MAPPPD for data for model

# Keep all positive adelie counts from 1982-2015 (ASSUMPTION 1) except one Avian Island count which has a very large discrepancy from the other counts at that site during similar time period 
# (ASSUMPTION 2) Keep maximum count for each site, season, (adult & nest)/chick combination
# Should be 1224 rows
sql <- "
select distinct maxcount.site_id, maxcount.season, maxcount.chick, adelielist.count_type, maxcount.count, adelielist.accuracy from
(select site_id, season, case when count_type = 'nests' or count_type = 'adults' then 0 else 1 end as chick, max(count) as count from preprocessed
where season >= 1982 and season <= 2015 and common_name='ad{\\''e}lie penguin' and count > 0 and not preprocessed_id = 192
group by site_id, season, chick) maxcount
inner join 
(select site_id, season, case when count_type = 'nests' or count_type = 'adults' then 0 else 1 end as chick, count_type, accuracy, count from preprocessed
where season >= 1982 and season <= 2015 and common_name='ad{\\''e}lie penguin' and count > 0 and not preprocessed_id = 192) adelielist
on maxcount.site_id = adelielist.site_id and maxcount.season = adelielist.season and maxcount.chick = adelielist.chick and maxcount.count = adelielist.count
order by site_id, season, chick;"
abundance <- dbGetQuery(con, sql)
dim(abundance)

# Keep all presence/absence data from 1982-2015 (ASSUMPTION 3) except for absence data made via vhr as this could be unreliable 
# Keep only sites that also have at least one adelie abundance count between 1982-2015
# Should be 1166 rows
sql <- "
select adelielist.site_id, adelielist.season, adelielist.presence from
(select distinct site_id from preprocessed
where season >= 1982 and season <= 2015 and count > 0 and common_name='ad{\\''e}lie penguin' and not preprocessed_id = 192) abundancelist
inner join 
(select site_id, season, max(presence) as presence from preprocessed
where season >= 1982 and season <= 2015 and common_name='ad{\\''e}lie penguin' and not (vantage = 'vhr' and presence = 0) and not preprocessed_id = 192
group by site_id, season) adelielist
on abundancelist.site_id = adelielist.site_id
order by site_id, season;"
occupancy <- dbGetQuery(con, sql)
dim(occupancy)

# Keep only sites that have at least one adelie abundance count between 1982-2015
# Should be 267 sites
sql <- "
select sitelist.site_id, sitelist.site_name, sitelist.region, sitelist.ccamlr_id from
(select distinct site_id from preprocessed
where season >= 1982 and season <= 2015 and count > 0 and common_name='ad{\\''e}lie penguin') abundancelist
inner join 
(select site_id, site_name, region, ccamlr_id from site) sitelist
on abundancelist.site_id = sitelist.site_id
order by site_id;"
SiteList <- dbGetQuery(con, sql)
dim(SiteList)

# Keep only covariates for sites that have at least one adelie abundance count between 1982-2015
# Should be 18156 rows, 267 sites x 34 years x 2 covariates
sql <- "
select covlist.site_id, covlist.season, covlist.month_start, covlist.month_stop, covlist.lag_start, covlist.lag_stop, covlist.statistic, covlist.radius, covlist.value from
(select distinct site_id from preprocessed
where season >= 1982 and season <= 2015 and count > 0 and common_name='ad{\\''e}lie penguin') abundancelist
inner join 
(select site_id, season, radius, statistic, month_start, month_stop, lag_start, lag_stop, value from sitecovariates
where season >= 1982 and season <= 2015 and statistic = 'max' and ((radius = 500 and month_start = 6 and month_stop = 9 and lag_start = 0 and lag_stop = 4)
or (radius = 500 and month_start = 11 and month_stop = 1 and lag_start = 4 and lag_stop = 4))) covlist
on abundancelist.site_id = covlist.site_id
order by site_id, season;"
sitecov <- dbGetQuery(con, sql)
dim(sitecov)

###################################################################################################################
# Merge pre-determined observations to drop for data validation purposes
# perform no data validation this run

 dim(abundance)
 DataValidationRows <- read.csv("DataValidationRows.csv", header = TRUE)
 abundance <- merge(abundance, DataValidationRows, by = c("site_id", "season", "count", "count_type", "accuracy"), all.x = TRUE)
 dim(abundance)
 i <- which(is.na(abundance$validation==TRUE)); abundance$validation[i] <- 0

abundance$validation <- 0

###################################################################################################################
# Some more assumptions

# (ASSUMPTION 4) We convert adults counts to nest counts 1:1, while increasing the error by + 3 with a max error of 5 for such counts
adults <- which(abundance$count_type == "adults")
for (i in 1:length(adults)) {
  abundance$accuracy[adults[i]] <- min(abundance$accuracy[adults[i]] + 3, 5)
}

# (ASSUMPTION 5) convert accuracy to the following errors/precisions, see supplement 1 for details regarding this
abundance$precision <- NA
i <- which(abundance$accuracy == 1); abundance$precision[i] <- 1/0.02490061^2
i <- which(abundance$accuracy == 2); abundance$precision[i] <- 1/0.04955838^2
i <- which(abundance$accuracy == 3); abundance$precision[i] <- 1/0.1201131^2
i <- which(abundance$accuracy == 4); abundance$precision[i] <- 1/0.2212992^2
i <- which(abundance$accuracy == 5); abundance$precision[i] <- 1/0.4472728^2

###################################################################################################################
# make sea ice sitecovariates wide not long

sitecov2 <- subset(sitecov, month_start==11 & month_stop==1 & lag_start==4 & lag_stop==4, select = c(site_id, season, value))
names(sitecov2) <- c("site_id", "season", "sic_11_1_max_l4_500")
sitecov3 <- subset(sitecov, month_start==6 & month_stop==9 & lag_start==0 & lag_stop==4, select = c(site_id, season, value))
names(sitecov3) <- c("site_id", "season", "sic_6_9_max_l0_l4_500")
sitecov4 <- merge(sitecov2, sitecov3, by = c("site_id", "season"))
sitecov <- subset(sitecov4, select = c("site_id", "season", "sic_11_1_max_l4_500", "sic_6_9_max_l0_l4_500"))

###################################################################################################################
# Indexing

# Construct site index
abundance$site <- as.numeric(as.factor(abundance$site_id))
occupancy$site <- as.numeric(as.factor(occupancy$site_id))
SiteList$site <- as.numeric(as.factor(SiteList$site_id))
SiteList$ccamlr <- as.numeric(as.factor(SiteList$ccamlr_id))
sitecov$site <- as.numeric(as.factor(sitecov$site_id))

# Construct relative season index
min_season <- 1982
abundance$season_relative <- abundance$season - min_season + 1
occupancy$season_relative <- occupancy$season - min_season + 1
sitecov$season_relative <- sitecov$season - min_season + 1

# Compute visit index
abundance$visit <- 1
for (i in 2:dim(abundance)[1]) {
  if (abundance$site[i] == abundance$site[i - 1] & abundance$season[i] == abundance$season[i - 1]) abundance$visit[i] <- abundance$visit[i - 1] + 1
}

# Compute the number of sites
(n_sites <- length(unique(abundance$site)))

# Compute the number of seasons
(n_seasons <- (max(abundance$season) - min_season) + 2)

# Compute the number of counts
abundance_observed <- subset(abundance, validation==0)
abundance_predict <- subset(abundance, validation==1)
(n <- dim(abundance_observed)[1])
# (n_p <- dim(abundance_predict)[1])

# These vectors allow the model to select only the non-NA non-validation 1982 - 2015 tuples from the data cubes created below for estimation
(site <- as.vector(abundance_observed$site))
(season <- as.vector(abundance_observed$season_relative))
(visit <- as.vector(abundance_observed$visit))

# These vectors allow the model to select only the non-NA validation tuples from the data cubes created below for validation
# (site_p <- as.vector(abundance_predict$site))
# (season_p <- as.vector(abundance_predict$season_relative))
# (visit_p <- as.vector(abundance_predict$visit))

# Compute the initial and final seasons that we have count data for each site
abundance2 <- tbl_df(abundance) %>% 
  group_by(site) %>%
  summarise('initial' = min(season_relative), 'final' = max(season_relative))

# This vector allows the model to find the initial year of data for each site
(initial <- as.vector(abundance2$initial))
dim(abundance); abundance <- merge(abundance, abundance2, by = "site"); dim(abundance)

###################################################################################################################
# Some more assumptions

# (ASSUMPTION 6) If the initial year count is a chick count we convert chicks to nests using the model hyperpriors

i <- which(abundance$initial==abundance$season_relative & abundance$count_type=="chicks" & abundance$visit==1)
length(i)
abundance$count_type[i] <- "nests"
abundance$chick[i] <- 0
abundance$count[i] <- round(abundance$count[i] / .90)

###################################################################################################################
# Extend SIC covariates as five year average in order to make predictions in 2015, or t + 1 
# This step won't be necessary once we can query MAPPPD for 2015 SIC 
# Scale covariates

sitecov2 <- tbl_df(sitecov) %>% 
  filter(season >= 2011, season <= 2015) %>% 
  group_by(site_id, site) %>%
  summarise('sic_6_9_max_l0_l4_500' = mean(sic_6_9_max_l0_l4_500), 'sic_11_1_max_l4_500' = mean(sic_11_1_max_l4_500)) %>%
  mutate(season = 2016, season_relative = n_seasons)

sitecov3 <- tbl_df(sitecov) %>% 
  filter(season < 2016)  %>% 
  select(site_id, season, site, season_relative, sic_6_9_max_l0_l4_500, sic_11_1_max_l4_500)

sitecov4 <- as.data.frame(rbind(as.data.frame(sitecov2), as.data.frame(sitecov3)))

sitecov5 <- sitecov4[order(sitecov4$site_id, sitecov4$season), ]

wsicMean <- mean(sitecov5$sic_6_9_max_l0_l4_500)
wsicSD <- sd(sitecov5$sic_6_9_max_l0_l4_500)
ssicMean <- mean(sitecov5$sic_11_1_max_l4_500)
ssicSD <- sd(sitecov5$sic_11_1_max_l4_500)

sitecov5$sic_6_9_max_l0_l4_500 <- as.numeric(scale(sitecov5$sic_6_9_max_l0_l4_500))
sitecov5$sic_11_1_max_l4_500 <- as.numeric(scale(sitecov5$sic_11_1_max_l4_500))

###################################################################################################################
# Create the data list for the JAGS model

# Make data cubes y, precision, and chick with dimensions [n_sites, n_seasons, 2] and matrices w and check with dimensions [n_sites, n_seasons]
chick <- y <- precision <- array(NA, dim = c(n_sites, n_seasons, 2))
check <- array(0, dim = c(n_sites, n_seasons, 2))
w <- array(NA, dim = c(n_sites, n_seasons))

for (i in 1:n_sites) {
  for (j in 1:n_seasons) {
    x1 <- subset(occupancy, site == i & season_relative == j)
    if (dim(x1)[1] > 0) {
      w[i, j] <- x1$presence
    }
    for (k in 1:2) {
      x2 <- subset(abundance, site == i & season_relative == j & visit == k)
      if (dim(x2)[1] > 0) {
        y[i, j, k] <- x2$count
        precision[i, j, k] <- x2$precision
        chick[i, j, k] <- x2$chick
        check[i, j, k] <- dim(x2)[1]
      }
    }
  }
}

# (ASSSUMPTION 7) We assume no colonization for any site from 1982 - 2014 and that all sites were colonized in 1982 
# This allows us to fill in NAs between presence counts and between the sart year and the first presence count with 1's
for (i in 1:n_sites) {
  for (j in n_seasons:2) {
    if (is.na(w[i, j]) == FALSE) {
      if (w[i, j] == 1) 
        w[i, (j - 1)] <- 1
    }
  }
}

# Make matrices for covariates with dimensions [n_sites, n_seasons]
sic_6_9_max_l0_l4_500 <- sic_11_1_max_l4_500 <- array(NA, dim = c(n_sites, n_seasons))

for (i in 1:n_sites) {
  for (j in 1:n_seasons) {
    x3 <- subset(sitecov5, site == i & season_relative == j)
    sic_6_9_max_l0_l4_500[i, j] <- x3$sic_6_9_max_l0_l4_500
    sic_11_1_max_l4_500 [i, j] <- x3$sic_11_1_max_l4_500 
  }
}

# The max check should be 1, meaning the subsetting only found one row per site, season, and visit
max(check)

DataQuery <- list(
  y = y, 
  w = w,
  n_sites = n_sites,
  n_seasons = n_seasons,
  initial = initial,
  n = n,
  site = site,
  season = season,
  visit = visit,
  precision = precision,
  chick = chick,
  wsic = sic_6_9_max_l0_l4_500,
  ssic = sic_11_1_max_l4_500)
#  n_p = n_p,
#  site_p = site_p,
#  season_p = season_p,
#  visit_p = visit_p)

# These frames are needed for generating the supplement
abundance5 <- tbl_df(abundance) %>% 
  filter(season <= 2016) %>%
  group_by(site) %>%
  summarise('initial' = min(season_relative), 'final' = max(season_relative))
SiteList <- merge(SiteList, abundance5, by = "site")
SiteList$seasons <- SiteList$final - SiteList$initial + 1

yframe <- abundance

StandardizationValues <- list(
  wsicMean = wsicMean,
  wsicSD = wsicSD,
  ssicMean =ssicMean,
  ssicSD <- ssicSD)

# Determine number of observed colonizaton and extinction events

dat <- data.frame(w)
dat.new <- do.call(rbind, lapply(1:nrow(dat), function(x) t(matrix(dat[x, order(is.na(dat[x,]))]))))
colnames(dat.new) <- colnames(dat)
dat.new <- data.frame(dat.new)
dat.new[is.na(dat.new)] <- 5

colonizations <- extinctions <- rep(0, dim(dat.new)[1])

for(i in 1:dim(dat.new)[1]){
  for(j in 2:dim(dat.new)[2]){
    if (dat.new[i, (j-1)]==1 & dat.new[i, j]==0) extinctions[i] <- extinctions[i] + 1
    if (dat.new[i, (j-1)]==0 & dat.new[i, j]==1) colonizations[i] <- colonizations[i] + 1
  }  
}  

table(colonizations)
table(extinctions)

keep <- c("DataQuery", "SiteList", "yframe", "StandardizationValues")
ls()[!(ls() %in% keep)]
rm(list = ls()[!(ls() %in% keep)])

save(DataQuery, file = "DataQuery.rda")
save(SiteList, file = "SiteList.rda")
save(yframe, file = "yframe.rda")
save(StandardizationValues, file = "StandardizationValues.rda")
```
