# Supplementary material for: Pan-Antarctic analysis aggregating spatial estimates of Adélie penguin abundance reveals robust dynamics despite stochastic noise
Source: Nat Commun. 2017 Oct 10;8:832. doi: 10.1038/s41467-017-00890-0 (PMC5635117; doi:10.1038/s41467-017-00890-0)
Supplement: Supplementary file 6 — Supplementary Data 4 [file 41467_2017_890_MOESM6_ESM.html]

#### Supplementary Data 4

Analysis of correlations between the stochastic year-effects from the Adélie population model (ver. 1.2 in www.penguinmap.com) and environmental covariates.

---

#### Table of Contents

I. Introduction

II. Year effects

III. Interdecadal Pacific Oscillation (IPO)

IV. Southern Oscillation Index (SOI)

V. Southern Annular Mode (SAM)

VI. Niño 3.4 (ENSO)

VII. Sea surface temperature

VIII. Sea ice concentration

IX. Sea surface winds

X. References

---

#### I. Introduction

We used cross-correlation analysis1 to explore relationships between the year effects estimated in the Adélie population model and various climatic indices linked to environmental conditions that potentially may affect Adélie population dynamics. We report the auto-correlation function (ACF) for the stochastic year effects and all climatic indices, as well as the cross-correlation function (CCF) between the year effects and each index. The cross-correlation coefficient at lag \(n\) is calculated as:

\[\textrm{CCF}\_{n}=\cfrac{\textrm{Cov}\big(\textrm{climate index}, \textrm{year effects}\big)}{SD\_{\textrm{climate index}}SD\_{\textrm{year effects}}},\]

where the biologically relevant lags are \(n=-12,\ldots,0\), reflecting changes in climate conditions that precede changes in Adélie population growth rate within a single Adélie generation. Note that in the case of sea surface temperature, sea ice concentration, and sea surface winds we restricted our analysis to 5 year lags. The 95% confidence interval for the ACF or CCF is either \(\pm \, ^2 / \_\sqrt{35} \approx \pm \, 0.33\) or \(\pm \, ^2 / \_\sqrt{34} \approx \pm \, 0.34\) depending on the length of the time series. These intervals are interpreted as an interval that would contain 95% of the correlation coefficients between two time series (or a time series and itself) of length 34 or 35 with zero correlation. Note, these confidence intervals have not been corrected for the number of statistical hypotheses being tested, and weakly significant results are likely due to the high family-wise type I error rate inherent to our analysis. Also, it is difficult to interpret these results as causal links between climate indices and population growth rates are likely to be site- or region-specific. A full investigation of this phenomena is outside the scope of our analysis.

All climate data was obtained at monthly intervals, and we performed this analysis by averaging these values at three temporal scales:

1. The early-breeding season (October - December), during which birds arrive at the colony and incubate eggs which subsequently hatch in late December2.
2. The chick rearing period (January - March), during which adults feed chicks until they fledge2.
3. All months during the winter and spring prior to the breeding season (April - September), during which colonies are unoccupied and birds are at sea2.

We used the means of the posterior distributions as point estimates for the year effects in this analysis.

#### II. Year effects

Fig. S4-1: Year effects \(\epsilon\_{y}\). Thick lines represent the 50% equal-tailed credible intervals, thin lines represent the 95% equal-tailed credible intervals, and circles are the posterior medians.

Fig. S4-2: Correlogram of year effects. The blue dotted line represents the 95% confidence interval of \(\pm \, 0.35\) under the null hypothesis of no correlation.

Table S4-1: Year effects \(\epsilon\_{y}\) posterior means and standard deviations.

| Start Year (Season) | End year | Mean | Standard Deviation |
| --- | --- | --- | --- |
| 1982 | 1983 | -1.96E-03 | 1.10E-01 |
| 1983 | 1984 | 3.16E-02 | 6.00E-02 |
| 1984 | 1985 | 2.30E-02 | 4.94E-02 |
| 1985 | 1986 | 8.08E-02 | 4.84E-02 |
| 1986 | 1987 | 4.77E-02 | 4.75E-02 |
| 1987 | 1988 | 3.79E-02 | 4.90E-02 |
| 1988 | 1989 | -6.57E-02 | 4.92E-02 |
| 1989 | 1990 | -1.39E-01 | 4.93E-02 |
| 1990 | 1991 | -1.76E-01 | 5.30E-02 |
| 1991 | 1992 | 1.19E-02 | 4.93E-02 |
| 1992 | 1993 | 7.04E-02 | 4.75E-02 |
| 1993 | 1994 | -8.43E-02 | 4.86E-02 |
| 1994 | 1995 | -1.39E-03 | 5.09E-02 |
| 1995 | 1996 | 8.44E-03 | 5.02E-02 |
| 1996 | 1997 | -9.60E-02 | 5.24E-02 |
| 1997 | 1998 | 1.00E-01 | 5.25E-02 |
| 1998 | 1999 | -5.53E-02 | 5.34E-02 |
| 1999 | 2000 | 9.22E-02 | 5.41E-02 |
| 2000 | 2001 | -9.04E-02 | 5.50E-02 |
| 2001 | 2002 | -1.09E-01 | 5.47E-02 |
| 2002 | 2003 | 1.41E-01 | 5.07E-02 |
| 2003 | 2004 | -1.39E-01 | 5.56E-02 |
| 2004 | 2005 | 1.19E-01 | 5.54E-02 |
| 2005 | 2006 | 8.86E-02 | 5.77E-02 |
| 2006 | 2007 | -1.69E-01 | 5.55E-02 |
| 2007 | 2008 | 9.48E-03 | 6.23E-02 |
| 2008 | 2009 | -3.40E-03 | 5.98E-02 |
| 2009 | 2010 | 1.48E-01 | 5.87E-02 |
| 2010 | 2011 | 2.23E-02 | 5.16E-02 |
| 2011 | 2012 | -8.30E-02 | 6.25E-02 |
| 2012 | 2013 | 7.18E-02 | 6.31E-02 |
| 2013 | 2014 | -1.86E-02 | 5.80E-02 |
| 2014 | 2015 | 5.09E-02 | 8.42E-02 |
| 2015 | 2016 | 5.89E-02 | 9.27E-02 |
| 2016 | 2017 | -1.46E-03 | 1.13E-01 |

#### III. Interdecadal Pacific Oscillation (IPO)

We used the unfiltered IPO index based on the Extended Reconstructed Sea Surface Temperature dataset (ERSST V4)3,4,5. This time series was created by the School of Earth Sciences at the University of Melbourne and made available online by the NOAA/OAR/ESRL Physical Sciences Division in Boulder, Colorado, USA6.

##### III-1. Winter months preceeding breeding (IPO)

Fig. S4-2: Year effects \(\epsilon\_{y}\) posterior means (blue) and the average IPO for winter months (April - September) preceding breeding (green).

Fig. S4-3: Correlogram of the average IPO for the winter months (April - September) preceding breeding. The blue dotted line represents the 95% confidence interval of \(\pm \, 0.35\) under the null hypothesis of no correlation.

Fig. S4-4: Cross-correlation function for year effects and the average IPO for the winter months (April - September) preceding breeding. The blue dotted line represents the 95% confidence interval of \(\pm \, 0.35\) under the null hypothesis of no correlation.

##### III-2. Early-breeding season (IPO)

Fig. S4-5: Year effects \(\epsilon\_{y}\) posterior means (blue) and the average IPO for the early-breeding season (October - December) (green).

Fig. S4-6: Correlogram of the average IPO for the early-breeding season (October - December). The blue dotted line represents the 95% confidence interval of \(\pm \, 0.35\) under the null hypothesis of no correlation.

Fig. S4-7: Cross-correlation function for year effects and the average IPO for the early-breeding season (October - December). The blue dotted line represents the 95% confidence interval of \(\pm \, 0.35\) under the null hypothesis of no correlation.

##### III-3. Chick rearing period (IPO)

Fig. S4-8: Year effects \(\epsilon\_{y}\) posterior means (blue) and the average IPO for the chick rearing period (January - March) (green).

Fig. S4-9: Correlogram of the average IPO for the chick rearing period (January - March). The blue dotted line represents the 95% confidence interval of \(\pm \, 0.35\) under the null hypothesis of no correlation.

Fig. S4-10: Cross-correlation function for year effects and the average IPO for the chick rearing period (January - March). The blue dotted line represents the 95% confidence interval of \(\pm \, 0.35\) under the null hypothesis of no correlation.

#### IV. Southern Oscillation Index (SOI)

We used the SOI index created by the Climatic Research Unit at the University of East Anglia and made available online by the NOAA Working Group on Surface Pressure7,8,9.

##### IV-1. Winter months preceeding breeding (SOI)

Fig. S4-11: Year effects \(\epsilon\_{y}\) posterior means (blue) and the average SOI for winter months (April - September) preceding breeding (green).

Fig. S4-12: Correlogram of the average SOI for the winter months (April - September) preceding breeding. The blue dotted line represents the 95% confidence interval of \(\pm \, 0.35\) under the null hypothesis of no correlation.

Fig. S4-13: Cross-correlation function for year effects and the average SOI for the winter months (April - September) preceding breeding. The blue dotted line represents the 95% confidence interval of \(\pm \, 0.35\) under the null hypothesis of no correlation.

##### IV-2. Early-breeding season (SOI)

Fig. S4-14: Year effects \(\epsilon\_{y}\) posterior means (blue) and the average SOI for the early-breeding season (October - December) (green).

Fig. S4-15: Correlogram of the average SOI for the early-breeding season (October - December). The blue dotted line represents the 95% confidence interval of \(\pm \, 0.35\) under the null hypothesis of no correlation.

Fig. S4-16: Cross-correlation function for year effects and the average SOI for the early-breeding season (October - December). The blue dotted line represents the 95% confidence interval of \(\pm \, 0.35\) under the null hypothesis of no correlation.

##### IV-3. Chick rearing period (SOI)

Fig. S4-17: Year effects \(\epsilon\_{y}\) posterior means (blue) and the average SOI for the chick rearing period (January - March) (green).

Fig. S4-18: Correlogram of the average SOI for the chick rearing period (January - March). The blue dotted line represents the 95% confidence interval of \(\pm \, 0.35\) under the null hypothesis of no correlation.

Fig. S4-19: Cross-correlation function for year effects and the average SOI for the chick rearing period (January - March). The blue dotted line represents the 95% confidence interval of \(\pm \, 0.35\) under the null hypothesis of no correlation.

#### V. Southern Annular Mode (SAM)

We used the monthly mean SAM (or AAO) index made available online by the NOAA/NWS Climate Prediction Center10.

##### V-1. Winter months preceeding breeding (SAM)

Fig. S4-20: Year effects \(\epsilon\_{y}\) posterior means (blue) and the average SAM for winter months (April - September) preceding breeding (green).

Fig. S4-21: Correlogram of the average SAM for the winter months (April - September) preceding breeding. The blue dotted line represents the 95% confidence interval of \(\pm \, 0.35\) under the null hypothesis of no correlation.

Fig. S4-22: Cross-correlation function for year effects and the average SAM for the winter months (April - September) preceding breeding. The blue dotted line represents the 95% confidence interval of \(\pm \, 0.35\) under the null hypothesis of no correlation.

##### V-2. Early-breeding season (SAM)

Fig. S4-23: Year effects \(\epsilon\_{y}\) posterior means (blue) and the average SAM for the early-breeding season (October - December) (green).

Fig. S4-24: Correlogram of the average SAM for the early-breeding season (October - December). The blue dotted line represents the 95% confidence interval of \(\pm \, 0.35\) under the null hypothesis of no correlation.

Fig. S4-25: Cross-correlation function for year effects and the average SAM for the early-breeding season (October - December). The blue dotted line represents the 95% confidence interval of \(\pm \, 0.35\) under the null hypothesis of no correlation.

##### V-3. Chick rearing period (SAM)

Fig. S4-26: Year effects \(\epsilon\_{y}\) posterior means (blue) and the average SAM for the chick rearing period (January - March) (green).

Fig. S4-27: Correlogram of the average SAM for the chick rearing period (January - March). The blue dotted line represents the 95% confidence interval of \(\pm \, 0.35\) under the null hypothesis of no correlation.

Fig. S4-28: Cross-correlation function for year effects and the average SAM for the chick rearing period (January - March). The blue dotted line represents the 95% confidence interval of \(\pm \, 0.35\) under the null hypothesis of no correlation.

#### VI. Niño 3.4 (ENSO)

We used the Niño 3.4 index based on the based on the Extended Reconstructed Sea Surface Temperature dataset (ERSST V4)3,4,5 made available online by the NOAA/NWS Climate Prediction Center11.

##### V-1. Winter months preceeding breeding (ENSO)

Fig. S4-29: Year effects \(\epsilon\_{y}\) posterior means (blue) and the average Niño 3.4 for winter months (April - September) preceding breeding (green).

Fig. S4-30: Correlogram of the average Niño 3.4 for the winter months (April - September) preceding breeding. The blue dotted line represents the 95% confidence interval of \(\pm \, 0.35\) under the null hypothesis of no correlation.

Fig. S4-31: Cross-correlation function for year effects and the average Niño 3.4 for the winter months (April - September) preceding breeding. The blue dotted line represents the 95% confidence interval of \(\pm \, 0.35\) under the null hypothesis of no correlation.

##### V-2. Early-breeding season (ENSO)

Fig. S4-32: Year effects \(\epsilon\_{y}\) posterior means (blue) and the average Niño 3.4 for the early-breeding season (October - December) (green).

Fig. S4-33: Correlogram of the average Niño 3.4 for the early-breeding season (October - December). The blue dotted line represents the 95% confidence interval of \(\pm \, 0.35\) under the null hypothesis of no correlation.

Fig. S4-34: Cross-correlation function for year effects and the average Niño 3.4 for the early-breeding season (October - December). The blue dotted line represents the 95% confidence interval of \(\pm \, 0.35\) under the null hypothesis of no correlation.

##### V-3. Chick rearing period (ENSO)

Fig. S4-35: Year effects \(\epsilon\_{y}\) posterior means (blue) and the average Niño 3.4 for the chick rearing period (January - March) (green).

Fig. S4-36: Correlogram of the average Niño 3.4 for the chick rearing period (January - March). The blue dotted line represents the 95% confidence interval of \(\pm \, 0.35\) under the null hypothesis of no correlation.

Fig. S4-37: Cross-correlation function for year effects and the average Niño 3.4 for the chick rearing period (January - March). The blue dotted line represents the 95% confidence interval of \(\pm \, 0.35\) under the null hypothesis of no correlation.

#### VII. Sea surface temperature

We used the 1200-km version of the Goddard Institute for Space Studies (GISS) Surface Temperature Analysis (GISTEMP) based on the Extended Reconstructed Sea Surface Temperature dataset (ERSST V4)3,4,5 made available online by the NOAA/OAR/ESRL Physical Sciences Division in Boulder, Colorado, USA12.

Fig. S4-38: Cross-correlation function for year effects and sea surface temperature for the winter months (AMJJAS) preceding breeding breeding, the early-breeding season (OND), and the chick rearing period (JFM).

#### VIII. Sea ice concentration

We used the bootstrap sea ice concentration from Nimbus-7 SMMR and DMSP SSM/I-SSMIS, V2 made available online by the NASA National Snow and Ice Data Center in Boulder, Colorado, USA13.

Fig. S4-39: Cross-correlation function for year effects and sea ice concentration for the winter months (AMJJAS) preceding breeding breeding, the early-breeding season (OND), and the chick rearing period (JFM).

#### IX. Sea surface winds

We used the ERA-Interim atmospheric reanalysis of 10m surface winds made available online by the NCAR/UCAR Research Data Archive Computational and Information Systems Lab in Boulder, Colorado, USA14.

Fig. S4-40: Cross-correlation function for year effects and surface wind (U-component) for the winter months (AMJJAS) preceding breeding breeding, the early-breeding season (OND), and the chick rearing period (JFM).

Fig. S4-41: Cross-correlation function for year effects and surface wind (V-component) for the winter months (AMJJAS) preceding breeding breeding, the early-breeding season (OND), and the chick rearing period (JFM).

#### X. References

1. Venables, W. N. & Ripley, B. D. *Modern Applied Statistics with S. Fourth Edition*. Springer-Verlag, New York, New York (2002).
2. Ainley, D. G., LeResche, R. E., & Sladen, W. J. L. *Breeding biology of the Adelie penguin*. (University of California Press, Berkeley, California, 1983).
3. Huang, B., *et al.* Extended Reconstructed Sea Surface Temperature version 4 (ERSST.v4): Part I. Upgrades and intercomparisons. *Journal of Climate* **28**, 911-930 (2015).
4. Liu, W., *et al.* Extended Reconstructed Sea Surface Temperature version 4 (ERSST.v4): Part II. Parametric and structural uncertainty estimations. *Journal of Climate* **28**, 931-951 (2015).
5. Huang, B., *et al.* Further Exploring and Quantifying Uncertainties for Extended Reconstructed Sea Surface Temperature (ERSST) Version 4 (v4). *Journal of Climate* **29**, 3119-3142 (2016).
6. Henley, B. J., *et al.* A Tripole Index for the Interdecadal Pacific Oscillation. *Climate Dynamics* **45**, 3077-3090 (2015).
7. Ropelewski, C. F. & Jones, P. D. An extension of the Tahiti-Darwin Southern Oscillation Index. *Monthly Weather Review* **115**, 2161-2165 (1987).
8. Allan, R. J., Nicholls, N., Jones, P. D., & Butterworth, I. J. A further extension of the Tahiti-Darwin SOI, early SOI results and Darwin pressure. *Journal of Climate* **4**, 743-749 (1991).
9. Können, G. P., Jones, P. D., Kaltofen, M. H., & Allan, R. J. Pre-1866 extensions of the Southern Oscillation Index using early Indonesian and Tahitian meteorological readings. *Journal of Climate* **11**, 2325-2339 (1998).
10. Mo, K. C. Relationships between Low-Frequency Variability in the Southern Hemisphere and Sea Surface Temperature Anomalies. *Journal of Climate* **13**, 3599-3610 (2000).
11. Trenberth, K. E. The Definition of El Niño. *Bulletin of the American Meteorological Society* **78**, 2771-2777 (1997).
12. Hansen, J., Ruedy, R., Sato, M. & Lo, K. Global surface temperature change. *Rev. Geophys.* **48**, RG4004 (2010).
13. Comiso J. C. Bootstrap sea ice concentrations from Nimbus-7 SMMR and DMSP SSM/I-SSMIS, version 2 [1979–2005]. NASA National Snow and Ice Data Center Distributed Active Archive Center. http://dx.doi.org/10.5067/J6JQLS9EJ5HU (2015).
14. ECMWF (European Centre for Medium-Range Weather Forecasts). ERA-Interim Project, Single Parameter 6-Hourly Surface Analysis and Surface Forecast Time Series. Research Data Archive at the National Center for Atmospheric Research, Computational and Information Systems Laboratory. https://doi.org/10.5065/D64747WN. (2012, updated monthly).
