# Supplementary material for: Pan-Antarctic analysis aggregating spatial estimates of Adélie penguin abundance reveals robust dynamics despite stochastic noise
Source: Nat Commun. 2017 Oct 10;8:832. doi: 10.1038/s41467-017-00890-0 (PMC5635117; doi:10.1038/s41467-017-00890-0)
Supplement: Supplementary file 7 — Supplementary Data 5 [file 41467_2017_890_MOESM7_ESM.html]

#### Supplementary Data 5

Density dependence among time series from the Adélie population model (ver. 1.2 in www.penguinmap.com).

---

#### Table of Contents

I. Introduction

II. Actual growth rate as a function of true abundance

III. Ratio of actual to predicted growth rate as a function of true abundance

---

#### I. Introduction

We investigated whether Adélie population growth rate multiplier or the ratio of actual to predicted growth rate multipliers as functions of true abundance provides evidence for density-dependence. We selected all sites that had at least five annual transitions where both years contained counts. Using the posterior medians of true abundance and predicted population growth rate multiplier from the Adélie population model (Supplement 1), we calculated actual growth rate,

\[\lambda\_{s,y} = \frac{z\_{s,y + 1}}{z\_{s,y}},\]

and the ratio of actual to predicted growth rate multipliers,

\[\delta\_{s,y} = \cfrac{\lambda\_{s,y}}{e^{\,\beta\_{1} \,+\, \beta\_{2}\textrm{wsic}\_{s,y} \,+\, \beta\_{3}\textrm{ssic}\_{s,y} \,+\, \epsilon\_{y}}},\]

for all selected site-year combinations. Our motivation for using the ratio of actual to predicted growth was to ensure that trends in peak sea ice were not responsible for decreasing growth at a site. Positive values for the log of this ratio indicate that populations actually grew better than predicted, while negative values indicated the opposite. We regressed logs of these quantities on logged true abundance and considered a statistically significant slope at the 5% level as possible evidence for density dependence.

#### II. Actual growth rate as a function of true abundance

Fig. S5-1: Scatterplots of logged true abundances \(z\*\) and the logged actual population growth rate multipliers \(\lambda\) for all years where we also had observed count data for both \(y+1\) and \(y\) by site. The dotted line represents the predicted logged actual population growth rate multiplier as a function of logged true abundance. The slope of this line is shown in the title and the asterisk indicates if that slope was significant at the 5% level.

#### III. Ratio of actual to predicted growth rate as a function of true abundance

Fig. S5-2: Scatterplots of logged true abundance \(z\*\) and the logged population growth rate multiplier ratios \(\delta\) for all years where we also had observed count data for both \(y+1\) and \(y\) by site. Positive values for this ratio indicate that populations actually grew better than predicted, while negative values indicated the opposite. The thick dotted line represents the predicted logged population growth rate multiplier ratio as a function of logged true abundance. The slope of this line is shown in the title and the asterisk indicates if that slope was significant at the 5% level.
