# Supplementary material for: Pan-Antarctic analysis aggregating spatial estimates of Adélie penguin abundance reveals robust dynamics despite stochastic noise
Source: Nat Commun. 2017 Oct 10;8:832. doi: 10.1038/s41467-017-00890-0 (PMC5635117; doi:10.1038/s41467-017-00890-0)
Supplement: Supplementary file 8 — Supplementary Data 6 [file 41467_2017_890_MOESM8_ESM.html]

#### Supplementary Data 6

Simulations of process noise and trend recovery.

---

#### Table of Contents

I. Introduction

II. R function

III. Simulation

---

#### I. Introduction

The purpose of this simulation is to see how well one can recover the intrinsic rate of growth for a population or group of populations with a specified process variance. First, we simulate annual abundance for a 10-year time series from a single population using a simple exponential growth model as the median of a lognormal distribution, whose scale parameter is \(\sigma\) where \(N\_{1}\) = 1000 individuals, as:

\[N\_{t+1} \sim \textrm{lognormal}\big(N\_{t}e^{r}, \sigma^{2}\big)\textrm{.}\]

We can recover \(r\) by regressing logged abundance on time since:

\[\log\big(N\_{t}\big) = \log\big(N\_{1}\big) + rt\textrm{.}\]

If the regression recovers the correct sign for \(r\) and the p-value for \(r\) is \(<\) 0.05 we score the test statistic \(z\) as 1 and otherwise 0. We can simulate this time series \(n\) times and calculate \(p\), the proportion of times we unambiguously recover the correct sign of the trend as:

\[p = \cfrac{\sum\_{i=1}^{n}z\_{i}}{n}\]

We now vary the length of the time series as well as the number of sites. For multiple sites we first sum abundances across sites for each year to create a single aggregate time series. We then repeat the process described above.

#### II. R function

Below is an R function that allows you to specify the intrinsic rate of growth, scale parameter, population(s) start size, time series range, number of sites, and number of simulations and returns a matrix of \(p\) values, where \(p\) is defined above.

```
trendRecovery <- function(r = 0.05, process = 0.3, start = 1000, yearStart = 10, 
    yearEnd = 33, sites = 25, simulations = 1000) {
    
    # this matrix contains the proportion of simulation results where the sign
    # is unambigously recovered
    prop <- matrix(NA, nrow = (yearEnd - yearStart + 1), ncol = sites)
    
    for (y in yearStart:yearEnd) {
        for (s in 1:sites) {
            counter <- NA
            for (i in 1:simulations) {
                siteAbundance <- matrix(NA, nrow = y, ncol = s)
                abundance <- matrix(NA, nrow = y)
                siteAbundance[1, ] <- start
                abundance[1] <- sum(siteAbundance[1, ])
                time <- 1:length(abundance)
                for (j in 2:length(abundance)) {
                  siteAbundance[j, ] <- rlnorm(s, log(siteAbundance[j - 1, ] * 
                    exp(r)), process)
                  abundance[j] <- sum(siteAbundance[j, ])
                }
                pval <- summary(lm(log(abundance) ~ time))[[4]][2, 4]
                beta <- summary(lm(log(abundance) ~ time))[[4]][2, 1]
                if (beta > 0 & pval < 0.05) 
                  counter[i] <- 1 else counter[i] <- 0
            }
            prop[(y - yearStart + 1), s] <- sum(counter)/simulations
        }
    }
    
    rownames(prop) <- seq(yearStart, yearEnd)
    colnames(prop) <- seq(1, sites)
    prop2 <- melt(prop)
    names(prop2) <- c("years", "sites", "proportion")
    prop2$r <- r
    prop2$process <- process
    return(prop2)
}
```

#### III. Simulation

Using this R function, we run this simulation for populations whose intrinsic rate of growth are 0.01, 0.05, and 0.1 and whose scale parameters are 0.2, 0.25, 0.3, and 0.35. We perform this simulation for 1 to 25 sites and time series ranging from 10 to 33 years in length. All populations start at 1000 individuals and we use 2000 simulation for each combination of \(r\) and \(\sigma\).

Fig. S6-1: Heat maps showing the proportion of times the sign of the true population growth rate \(r\) was unambiguously recovered from time series of varying lengths (years) summed across varying numbers of sites. For each site, abundance was simulated using a simple exponential growth model (with true population growth rate \(r\) and \(N\_{1}=1000\)) as the median of a lognormal distribution whose scale parameter was \(\sigma\).
