# Supplementary material for: Pan-Antarctic analysis aggregating spatial estimates of Adélie penguin abundance reveals robust dynamics despite stochastic noise
Source: Nat Commun. 2017 Oct 10;8:832. doi: 10.1038/s41467-017-00890-0 (PMC5635117; doi:10.1038/s41467-017-00890-0)
Supplement: Supplementary file 9 — Supplementary Data 7 [file 41467_2017_890_MOESM9_ESM.html]

#### Supplementary Data 7

Continental-wide model outcomes from the Adélie population model (ver. 1.2 in www.penguinmap.com).

---

#### Table of Contents

I. Data scarcity

II. Population trend

III. Population growth rates

IV. Breeding productivity

---

#### I. Data scarcity

Fig. S7-1: Histogram showing number of seasons of actual data for all 267 adélie sites in Antarctica. Note that over 45% of all sites have only a single census.

#### II. Population trend

Fig. S7-2: Continent-wide Adélie abundance \(z\*\). The gray (1982 - 2015) and green (2016) shaded area represents the 90% highest posterior density credible interval; the black line is the posterior median. Note that 2016 is beyond the end of our time series; all abundance estimates from 2016 reflect aggregated population forecasts from the model.

#### III. Population growth rates

Fig. S7-3: Map displaying the posterior medians of the average actual population growth rate multipliers for all 267 Adélie colonies. For each site, this was computed as the geometric mean of the ratios of abundance \(z\*\) in year \(y+1\) to year \(y\) for all years the site was occupied between 1982 and 2015.

Fig. S7-4: Map displaying the posterior medians of the average predicted population growth rate multipliers for all 267 Adélie colonies. For each site, this was computed as the geometric mean of the predicted growth rates (\(e^{r}\); see Equation 6, Supplement 1) for all years the site was occupied between 1982 and 2015.

Fig. S7-5: Average actual (red circles and black lines) and predicted (blue circles and orange lines) population growth rate multipliers for all 267 Adélie colonies (with CCAMLR subarea), ordered by magnitude. Thick lines represent the 50% equal-tailed credible intervals, thin lines represent the 95% equal-tailed credible intervals, and circles are the posterior medians. The population growth rate multipliers were computed as in Figs. S7-3 and S7-4, respectively.

#### IV. Breeding productivity

Fig. S7-6: Posterior medians of median breeding productivity \((\,\alpha\_{s, y}\,)\) for sites with chick counts. We have included the observed zero breeding productivity year for Litchfield Island in 2001 for completeness. This count was excluded from the Adélie model due to our choice of the lognormal distribution to model the observation process.
