# Supplementary material for: Pan-Antarctic analysis aggregating spatial estimates of Adélie penguin abundance reveals robust dynamics despite stochastic noise
Source: Nat Commun. 2017 Oct 10;8:832. doi: 10.1038/s41467-017-00890-0 (PMC5635117; doi:10.1038/s41467-017-00890-0)
Supplement: Supplementary file 10 — Supplementary Data 8 [file 41467_2017_890_MOESM10_ESM.html]

#### Supplementary Data 8

Site-specific nest abudances from the Adélie population model (ver. 1.2 in www.penguinmap.com) for the Antarctic Peninsula region (CCAMLR sub-areas 48.1 and 48.2).

---

#### Table of Contents

I. Data scarcity

II. Population trend

III. Sea ice conditions

IV. Site level plots

---

##### I. Data scarcity

Fig. S8-1: Histogram showing number of seasons of actual data for all 108 Adélie sites located in the Antarctic Peninsula region.

Fig. S8-2: Data matrix showing sites (columns) and years (rows) for all Adélie sites located in the Antarctic Peninsula region. White cells indicate no data, black cells indicate a nest count, red cells indicate a chick count, and yellow cells paired nest and chick count.

##### II. Population trend

Fig. S8-3: Adélie abundance \(z\*\) in the Antarctic Peninsula region. The gray (1982 - 2015) and green (2016) shaded area represents the 90% highest posterior density credible interval; the black line is the posterior median. Note that 2016 is beyond the end of our time series; all abundance estimates from 2016 reflect population forecasts from the model.

#### III. Sea ice conditions

Fig. S8-4: Average peak winter and summer sea ice concentration across all Adélie sites in the Antarctic Peninsula region. The gray shaded areas represent the 95% confidence intervals; the black lines are the means. Peak winter sea ice concentration is the maximum monthly concentration (June through September) in a 500 km radius around each Adélie site across the previous 5 winters. Peak summer sea ice concentration is the maximum monthly concentration (November through January) in a 500 km radius around each Adélie site four years prior. Confidence intervals less than zero were truncated to zero. Note that 2016 is beyond the end of our sea ice time series; all site-level peak winter (summer) sea ice concentrations in 2016 represent the average peak winter (summer) sea ice sea ice concentations from 2011 - 2015 at each site.

#### IV. Site level plots

For each Adélie site in the Antarctic Peninsula region, we show the posterior median and 75% highest posterior density credible interval for the average actual population growth rate multiplier. This was computed as the geometric mean of the ratios of abundance \(z\*\) in year \(y+1\) to year \(y\) for all years the site was occupied between 1982 and 2015. Note that in cases where Adélies have gone extinct at a site the population growth rate multiplier for that site reflects annual changes in nest abundance prior to extinction. We plot the annual Adélie nest abundance \(z\*\), nest (black circles) or chick (red circles) counts, and the seasons where no nests (black squares) or chicks (red squares) were observed. The gray (1982 - 2015) and green (2016) shaded areas represent the 75% highest posterior density credible intervals; the black linea are the posterior medians. The error bars represent the 90% highest posterior density credible intervals from the posterior predictive distributions for the nest or chick counts. Note that 2016 is beyond the end of our time series; all nest abundance estimates from 2016 reflect population forecasts from the model.
